# Preparation and pharmacokinetic study of mebendazole complex with HP-beta-cyclodextrin

**DOI:** 10.3389/fvets.2025.1611154

**Published:** 2025-08-07

**Authors:** Charles Ding, Yili Ding, Zhe Xu, Peini Wang, Shufeng Xu

**Affiliations:** ^1^College of Science, Mathematics and Technology, Wenzhou-Kean University, Wenzhou, China; ^2^Keck School of Medicine of USC, Los Angeles, CA, United States; ^3^Dorothy and George Hennings College of Science, Mathematics and Technology, Kean University, Union, NJ, United States; ^4^Life Science Department, Foshan University, Foshan, Guangdong, China

**Keywords:** mebendazole, HP-beta-cyclodextrin, inclusion complex, complex preparation, *in vitro* and *in vivo* pharmacokinetic study

## Abstract

Mebendazole, approved by the Food and Drug Administration (FDA) in 1974 to expel intestinal parasite infections, was found to exhibit multiple anti-cancer activities. However, due to its poor water solubility (0.33 ± 0.02 μg/mL), its clinical applications were greatly limited. Scientists at Wenzhou-Kean University have developed a formulation that could increase its water solubility as much as 18,333 times to reach 6.05 mg/mL as the best result to date. Through the complexation with HP-beta-cyclodextrin, 80% of mebendazole in the complex was released *in vitro* in 5 min, and only 20% of mebendazole was released under the same conditions for the pure drug, in a *in vivo* pharmacokinetic study conducted on dogs with a dose of 5 mg/kg. The *C*_max_ of mebendazole was increased from 8.96 ± 0.15 μg/mL to 17.34 ± 2.02 μg/mL. *T*_max_ of mebendazole was shortened from 12.00 ± 0.50 h to 10 ± 0.50 h. The half lift time was prolonged from 5.81 ± 0.36 h to 10.01 ± 2.07 h; the *AUC*_0-24_ was increased from 151.32 ± 5.92 μg·h/mL to 289.02 ± 15.83 μg·h/mL; and the bioavailability was improved by 91%, indicating that this complex can be pushed to the next step as an anti-tumor agent for its clinic practice.

## Introduction

Mebendazole is a synthetic benzimidazole carbamate that was approved by the Food and Drug Administration (FDA) in 1974 to expel intestinal parasite infections, hinder the formation of the microtubule system of parasite cells, make the cell cycle stop in the G2/M phase, and affect cell mitosis. Additionally, it inhibits the parasite’s ability to uptake glucose, causes the glycogen stored in the parasite’s body to deplete, and reduces the formation of adenosine triphosphate, ultimately interfering with energy metabolism and rendering the parasite un-survivable (1). Mebendazole is effective against soil-transmitted helminth infections in humans (2) caused by at least one type of nematodes (roundworms, hookworms, or whipworms).

Recently, mebendazole was demonstrated to exhibit preclinical efficacy against multiple cancers, including glioblastoma multiforme, medulloblastoma, and colon, breast, pancreatic, and thyroid cancers (3). It was considered as an excellent candidate for the treatment of brain tumors as a monotherapy and in combination with other treatments (4, 5). However, its poor water solubility (0.33 ± 0.02 μg/mL) hampered its performance and limited its clinical applications (6). Cancer drugs administered through IP or IV should have water solubility in the range of 5 mg/mL to 10 mg/mL to achieve the necessary therapeutic dose, and as an oral solid dosage formulation, mebendazole was used to treat advanced or metastatic gastrointestinal cancer with the dose of up to 4 g/day in the phase 2 clinic trial (3, 7). Patients with refractory gastrointestinal cancer were treated with individualized, dose-adjusted mebendazole. Therefore, there is an urgent need to find ways of increasing mebendazole’s water solubility.

Many methods such as particle size reduction (8), solid dispersion (9–18), nanosuspensions (19–24) and salt formation (25–28) have been used to improve mebendazole’s water solubility, and the results were not significant, more important, most of these methods involved the usage of polymers, which could be challenge for product’s quality control and scaled up. Based on this consideration, cyclodextrins are the ideal drug carriers for increasing mebendazole’s water solubility. The inclusion complexes of mebendazole with cyclodextrins such as α, β, γ, β-sulfated and HP-beta-cyclodextrins showed an increase in water solubility ranging from 16 to 31 times (29, 30), when heated with HP-beta-cyclodextrin in the presence of citric or tartaric acid at 95°C for 60 min. Mebendazole’s water solubility was increased to 0.68 mg/mL (31, 32), and the formulation of beta-cyclodextrin, chitosan-based microcrystals, polyvinyl alcohol and polysorbate 80-based nanoparticles could only improve mebendazole’s solubility four times (33). Complexes with beta-cyclodextrins and permethyl-beta-cyclodextrin could increase the solubility by 35 times and 4,700 times, respectively (34). It was reported that solid dispersion could increase mebendazole’s solubility 15,982 times (35); however, the solubility was measured in an acidic aqueous solution (0.1 M HCl), and therefore, the solubility result is not real.

**Figure 1:**
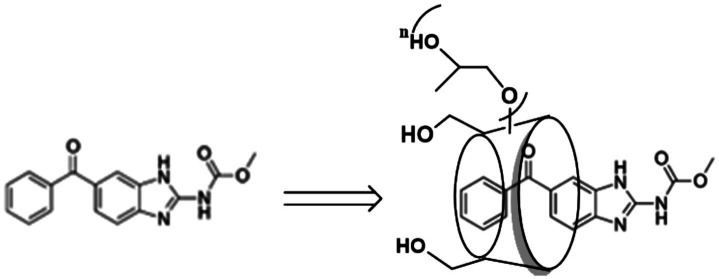
Structures of mebendazole and its complex with HP-beta-cyclodextrin.

Overall, although great efforts were made to increase mebendazole’s water solubility, a significant increase in water solubility was not achieved. In this communication, we report our updated results from a study of mebendazole’s water solubility through complexation with HP-beta-cyclodextrin. As shown in Figure 1, the water solubility of mebendazole increased 18,333 times to reach 6.05 mg/mL, which is the best result to date. Meanwhile, the pharmacokinetic properties of this complex were evaluated. Although HP-beta-cyclodextrin consists of a mixture with a specific range of molecular weight, the risk of the product quality control and scale-up was effectively mitigated.

## Results

### Establishment of the standard curve of mebendazole

A solution of mebendazole in formic acid with a concentration of 1 mg/mL was prepared and used for UV spectrum scanning in the range of 200–400 nm; the maximum absorption of mebendazole at 234 nm was determined and used for HPLC analysis with a UV detector.

**Figure 2:**
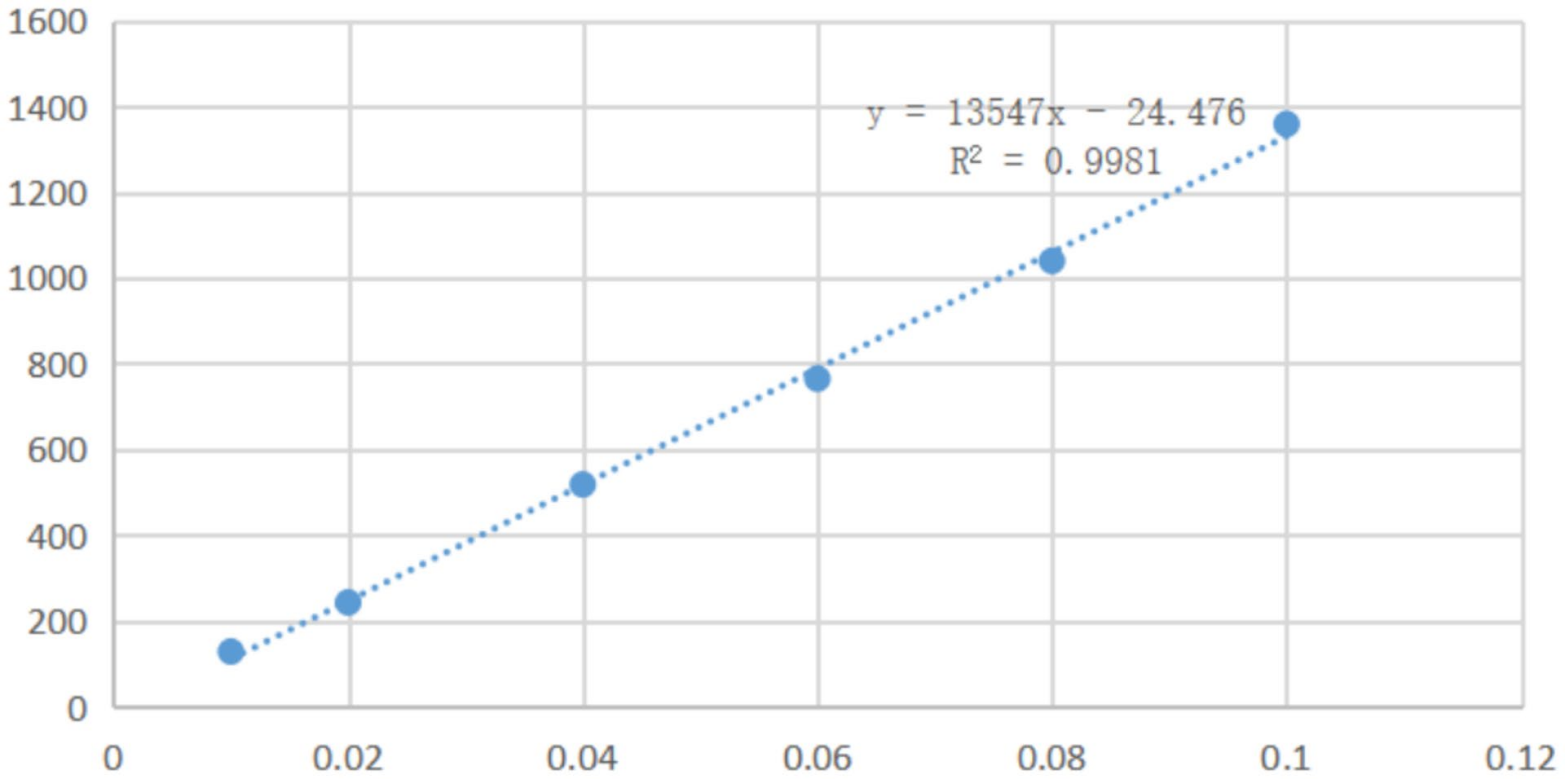
Standard curve and regression equation of mebendazole.

A series of solutions of mebendazole in a mixture of water and formic acid (10:1) with different concentrations were analyzed by HPLC with a UV detector at a wavelength of 234 nm. Based on the peak areas in the HPLC and the concentrations of mebendazole, it was found that, in the range of 0.01–0.1 mg/mL, the peak areas in HPLC were linearly related to the concentrations of mebendazole, and the linear regression equation was obtained as *Y* = 13,547X-24.476 (determination coefficient *R*^2^: 0.9981, Y: absorption peak area, X: concentration). The standard curve is shown in Figure 2.

### Phase solubility study

**Figure 3:**
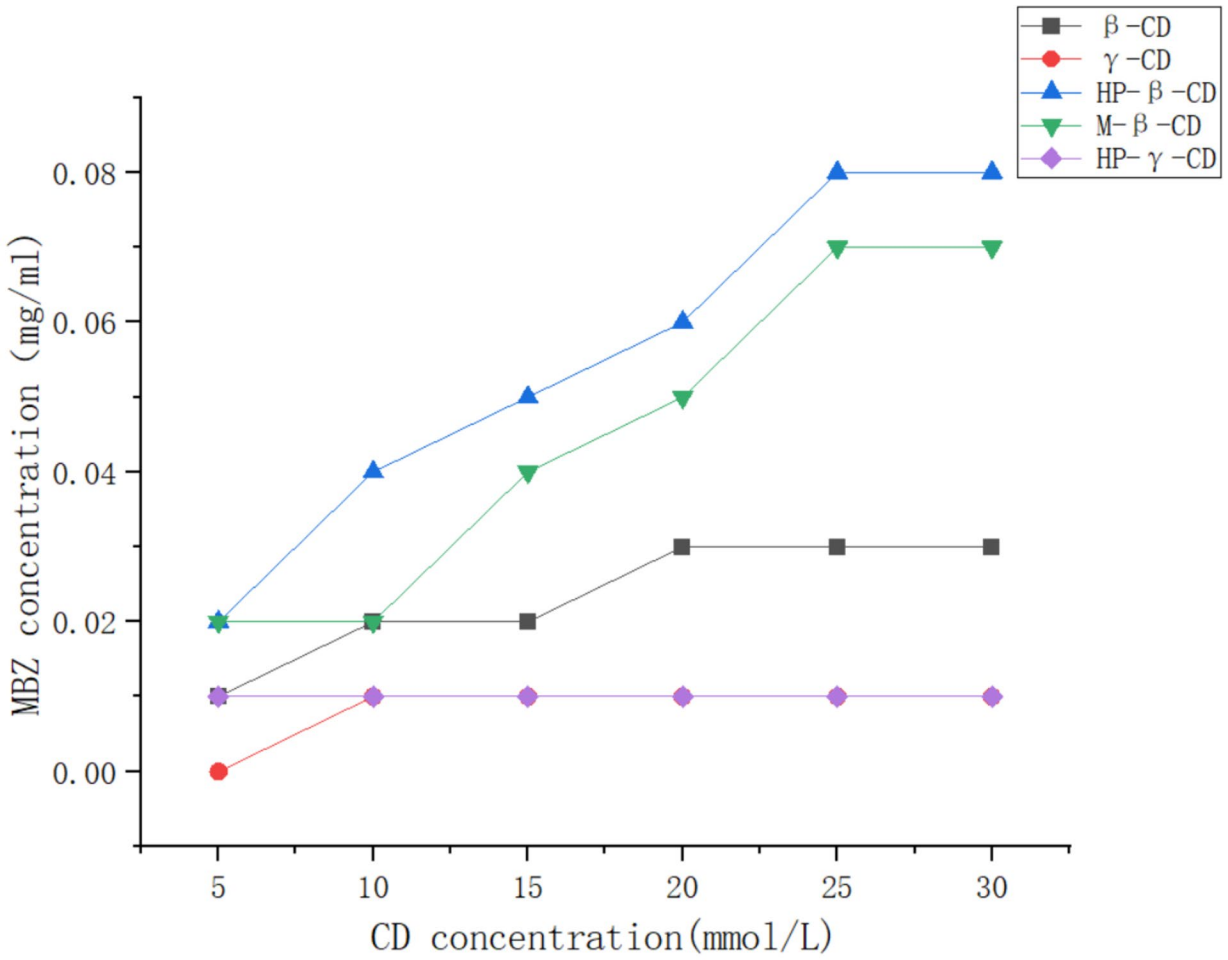
Solubilities of Mebendazole in the solutions of β-cyclodextrin, γ-cyclodextrin, methyl-β-cyclodextrin, HP-β-cyclodextrin, and HP-γ-cyclodextrin in water with different concentrations.

The solutions (10 mL) of β-cyclodextrin, γ-cyclodextrin, methyl-β-cyclodextrin, HP-β-cyclodextrin, and HP-γ-cyclodextrin in water with different concentrations were added to mebendazole and stirred at a speed of 200 r/min in a shaker at 25°C in the dark for 48 h. The selection of the concentrations of cyclodextrin derivatives for the solubility phase study was based on the solubilities of cyclodextrins. Each of the reaction mixtures (1 mL) was filtered and analyzed using HPLC. Based on the regression equation and standard curve, the amount of mebendazole in each solution was determined and summarized in Figure 3. The amounts of dissolved mebendazole in aqueous solutions of HP-beta-cyclodextrin and methyl-beta-cyclodextrin showed a linear increase in the range of 5 mmol/L to 25 mmol/L of the cyclodextrins, then plateaued and became independent of the cyclodextrin concentrations. The curves of HP-beta-cyclodextrin and methyl-beta-cyclodextrin can be considered as A_N_-type diagrams. The cavity of HP-beta-cyclodextrin exhibited better complex affinity than the others; the cavities of γ-cyclodextrin derivatives may be too large for mebendazole, beta-cyclodextrin has the solubility issue, and HP-beta-cyclodextrin may show better lipophilicity than methyl-beta-cyclodextrin. Therefore, HP-beta-cyclodextrin was selected to form a complex with mebendazole to prepare the complex.

### Preparation of the complex of mebendazole with cyclodextrins

Cyclodextrin complex conditions, including reaction time, reaction temperature, stirring speed, and the ratio between mebendazole and HP-beta-cyclodextrin, could affect the inclusion results. Based on single factors and orthogonal strategies, many complex conditions were tried, and water solubility was used as the criterion to judge the inclusion effect. The condition that was found to prepare the complex of mebendazole with HP-beta-cyclodextrin exhibited the best water solubility at 6.05 mg/mL so far, with 28% of inclusion rate and 90% of inclusion yield.

### Characterization of mebendazole complex

The complex was confirmed by SEM in its solid state and by NMR spectra in its liquid state, and used to study the PK properties.

**Figure 4:**
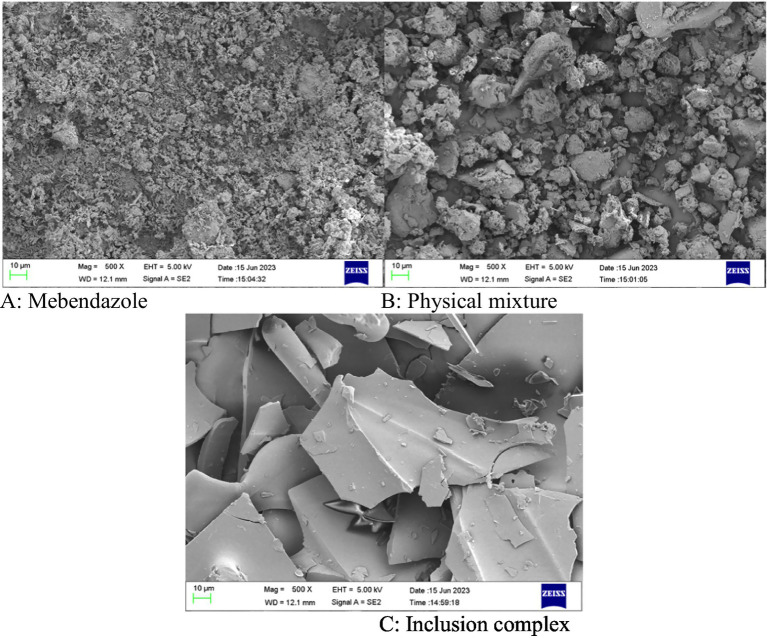
**(A–C)** The scanning electron microphotographs of mebendazole, the physical mixture of mebendazole and HP-beta-cyclodextrin, and the inclusion complex of mebendazole and HP-beta-cyclodextrin.

Mebendazole, the physical mixture of mebendazole and HP-beta-cyclodextrin, and their inclusion complex were checked by scanning electron microphotographs (Figure 4). Mebendazole shows fine crystals (A) that appear in the micrograph of the physical mixture (B), and is different from the microphotograph of the inclusion complex (C). The significant difference in the particle size and morphology of the inclusion complex from mebendazole crystals indicates the formation of a new phase.

**Figure 5:**
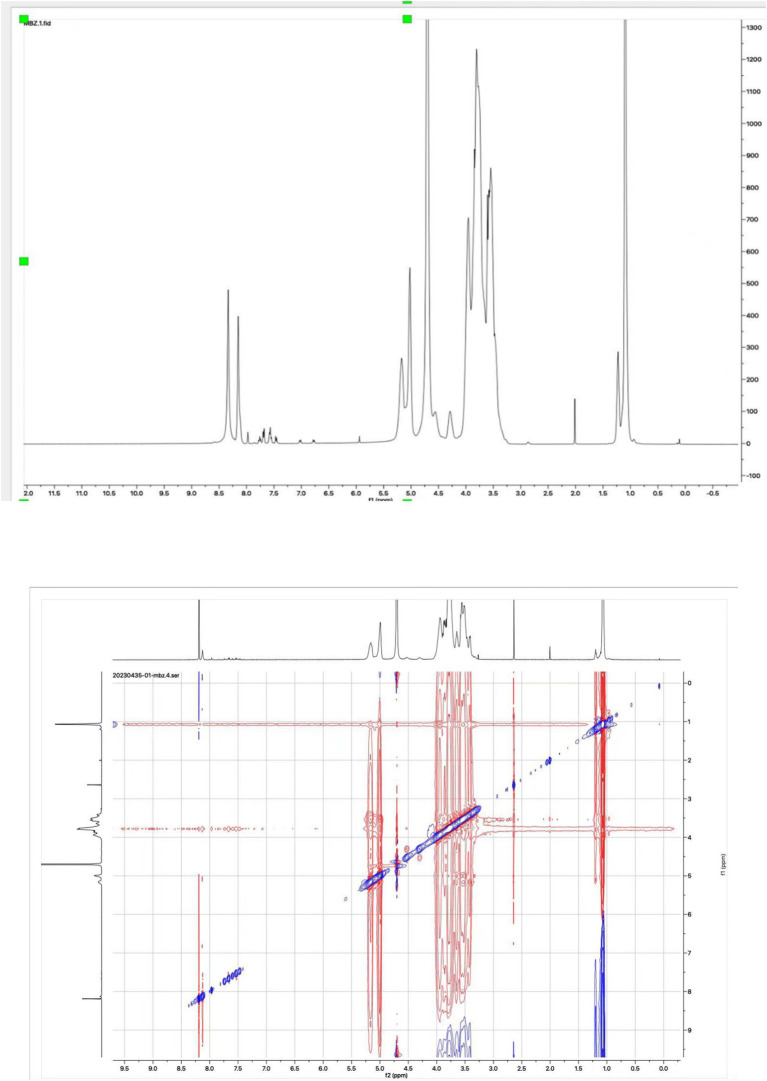
Proton NMR and ROESY spectra of the inclusion complex of Mebendazole with HP-beta-cyclodextrin in D_2_O.

NMR spectroscopy, as the most effective method, was used to confirm the formation of the mebendazole inclusion complex and the interaction between mebendazole and HP-beta-cyclodextrin in the complex. The 1D proton NMR and ROESY spectra of the inclusion complex in D_2_O were recorded and are shown in Figure 5. The signals at 7.97 ppm (1H, s), 7.74 ppm (1H, d), 7.63 ppm (2H, m), 7.50 ppm (3H, m) and 7.40 ppm (1H, d) in the proton NMR spectrum of the inclusion complex in D_2_O indicated that the complex contained the mebendazole molecules. The ROESY spectrum of the inclusion complex exhibited interactions between the protons at 7.97 ppm, 7.74 ppm, 7.63 ppm, 7.50 ppm, and 7.40 ppm from mebendazole and the protons at 3.80 ppm from HP-beta-cyclodextrin, indicating that mebendazole was in the cavity of HP-beta-cyclodextrin.

### The dissolution rate study

**Figure 6:**
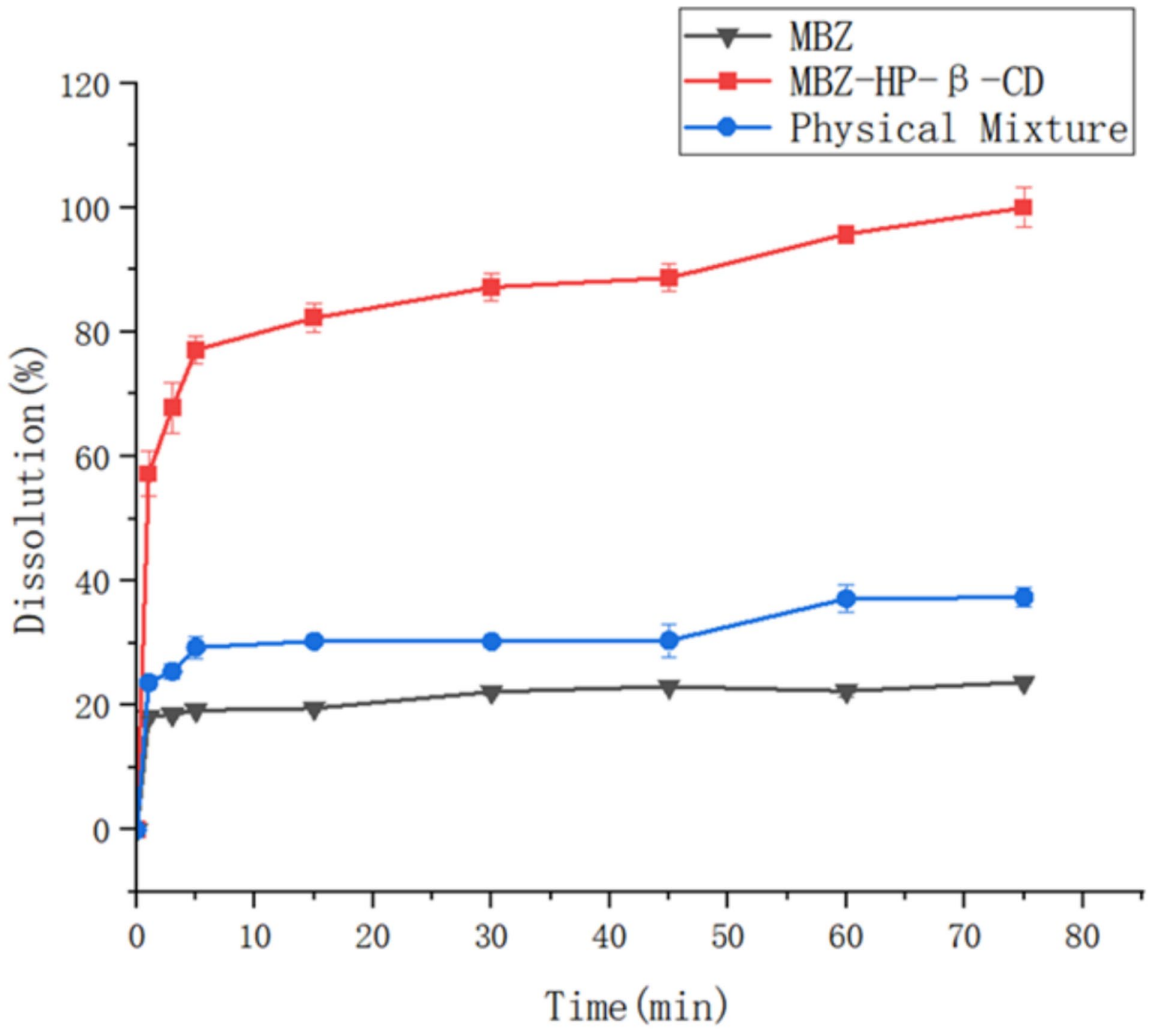
The dissolution rate curves of mebendazole, the physical mixture of Mebendazole and HP-beta-cyclodextrin, and their inclusion complex.

Mebendazole, the physical mixture of mebendazole and HP-beta-cyclodextrin, and the inclusion complex of mebendazole and HP-beta-cyclodextrin were used to study the dissolution rate, each experiment was repeated three times, with the standard deviations 0.01 to 0.09 for mebendazole, 0.15 to 0.42 for the complex, and 0.04 to 0.26 for the physical mixture, the results are shown in Figure 6.

### *In vivo* pharmacokinetic study

**Figure 7:**
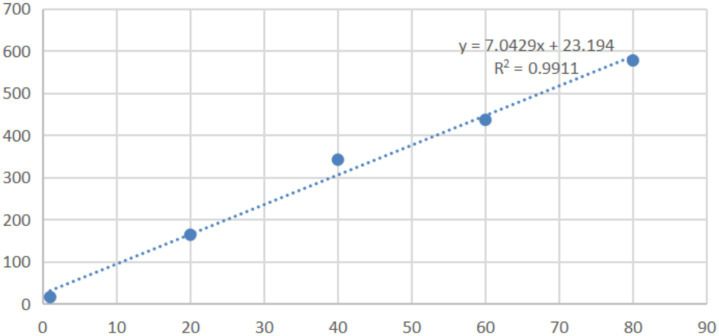
Standard curve and regression equation of mebendazole in blank dog plasma.

In the range of 0.005 μg/mL to 80 μg/mL, the peak areas in HPLC and the concentrations of mebendazole in blank dog plasma showed the linear relationship, the dog plasma did not interfere the detection of mebendazole, and the standard curve and regression equation (*Y* = 7.0429X + 23.194, *R*^2^ = 0.9911) of mebendazole in dog plasma were obtained and shown in Figure 7 with LLOD of 0.005 μg/mL and LLOQ of 0.015 μg/mL.

Twelve dogs (5 ± 0.1 kg), with half male and half female, were randomly divided into two groups as complex group and drug group, numbered and orally administered mebendazole or its inclusion complex at a dose of 5 mg/kg. The blood samples were collected by using heparinized tubes, centrifuged, and kept in a − 20°C freezer. The thawed plasma sample was extracted with organic solvents, centrifuged, filtered through a 0.22 μm microporous filter membrane, and analyzed by HPLC at room temperature.

The HPLC analysis of the plasma samples was conducted in a short time by using the same HPLC instrument and C-18 column; each time, before and after the HPLC analysis, the standard sample of mebendazole was checked for calibration to avoid system errors. The plasma samples were analyzed by HPLC.

**Figure 8:**
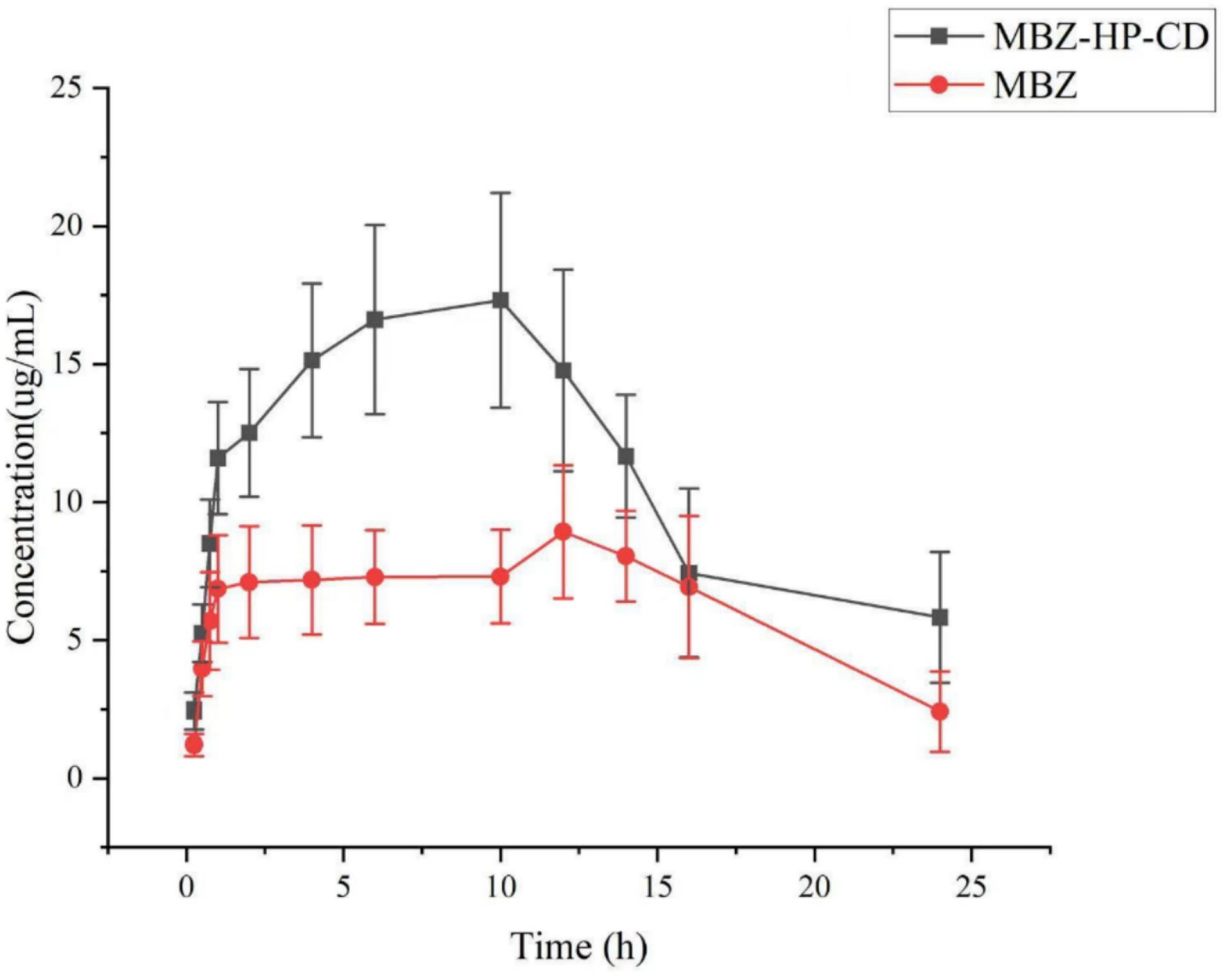
Curves of mebendazole concentrations in plasma from dogs administered with mebendazole or its HP-beta-cyclodextrin complex.

Based on the obtained HPLC data, the standard curve of mebendazole in plasma and the regression equation, the relationship between the concentration of mebendazole in plasma from dogs dosed with mebendazole or its inclusion complex and the collection time was obtained to produce the drug-time curve shown in Figure 8. The PK parameters were obtained by processing the data using WinNolin software, and the main pharmacokinetic parameters are summarized in Table 1.

**Table 1:** Pharmacokinetic parameters of icariin and its HP-beta-cyclodextrin complex.

Parameters	Unit	Value	
		Mebendazole	Mebendazole/HP-beta-cyclodextrin
*T*_max_	H	12.00 ± 0.50	10 ± 0.50
*C*_max_	μg/mL	8.96 ± 0.15	17.34 ± 2.02
AUC_0-24_	μg·h/mL	151.32 ± 5.92	289.02 ± 15.83
*t*_1/2_	H	5.81 ± 0.36	10.01 ± 2.07
MRT	H	13.35 ± 0.30	16.85 ± 0.22

## Discussion

Many methods can be used to confirm the formation of inclusion complexes of mebendazole with cyclodextrin derivatives, such as FTIR, DSC, TAG, X-ray, SEM, and mass spectra; however, these methods are not accurate and have limited correlation with mebendazole’s water solubility. In the majority of cases, the main purpose of preparing the inclusion complex of mebendazole is to increase its water solubility. NMR spectra is the most powerful tool that not only confirms the interactions between mebendazole and cyclodextrin derivatives in the complex but also indicates the water solubility of mebendazole in the complex when D_2_O was used as the solvent.

In 2007, mebendazole was complexed with different cyclodextrins, including α-cyclodextrin, β-cyclodextrin, γ-cyclodextrin, HP-α-cyclodextrin, HP-β-cyclodextrin, HP-γ-cyclodextrin, and permethyl beta-cyclodextrin in a mixture of acetone or acetonitrile and water, with or without formic acid. Permethyl beta-cyclodextrin could increase the water solubility of mebendazole by 4,700 times (34). The same result was reported in 2008 (36), and the compound permethyl beta-cyclodextrin was defined as partially methylated beta-cyclodextrin. The mass spectrum of the complex was recorded, but the mass peak for the complex (mebendazole + permethyl beta-cyclodextrin) was not detected; the NMR spectrum of the complex in D_2_O was not recorded. In the present study, the complex of mebendazole with methyl beta-cyclodextrin was confirmed by proton NMR and ROESY spectra in D_2_O.

In 1999, the NOESY spectrum of mebendazole and *γ*-cyclodextrin or *α*-cyclodextrin was recorded (30), and the NOESY signals indicated the interaction between the mebendazole and cyclodextrins. However, the NMR spectrum was recorded in DMSOd_6_, which indicated that the complex was formed in DMSO and had less relation to the drug’s water solubility. The proton NMR and ROESY spectra in D_2_O can not only confirm the formation of the complex, but also indirectly confirm the complex’s water solubility.

The inclusion complex of mebendazole and *citrate*-beta-cyclodextrin was prepared in the mixture of formic acid and water, but the water solubility of mebendazole was increased only four-fold (33). As mentioned in the introduction, the solubility increase of cyclodextrin complexations in other literature reports is in the range of 4-fold to 2020-fold, and the complexes and solubility were not confirmed by NMR spectra in D_2_O.

In summary, the formulation in this study showed the best mebendazole water solubility increasing so far. The solubility and interaction between mebendazole and HP-beta-cyclodextrin in the complex were confirmed by proton NMR and ROESY spectra in D_2_O.

The dissolution rate studies showed that 80% of mebendazole was released from the inclusion complex within 15 min, whereas under the same conditions, only 30% of mebendazole was released from its physical mixture, and only 20% was released from the pure mebendazole.

The *in vivo* PK study showed that through complexation with HP-*β*-cyclodextrin, *C*_max_ of mebendazole was increased from 8.96 ± 0.15 μg/mL to 17.34 ± 2.02 μg/mL, *T*_max_ of mebendazole was shorted from 12.00 ± 0.50 h to 10 ± 0.50 h, the half lift time was prolonged from 5.81 ± 0.36 h to 10.01 ± 2.07 h, the AUC_0-24_ was increased from 151.32 ± 5.92 μg·h/mL to 289.02 ± 15.83 μg·h/mL, and the bioavailability was increased 91%.

Oral medications are typically swallowed and absorbed in the stomach or small intestine before entering the portal venous system and passing through the liver, and then into the systemic circulation.

When the drug is taken orally, it is broken down by stomach acids before it passes through the liver and then enters the bloodstream. When the medication is coated or complexed with cyclodextrin derivatives, the drug is transferred into the stomach much more than the drug alone. It is not surprising that the *C*_max_ of mebendazole in the complex group is larger than the *C*_max_ in the pure drug group. The solubility of mebendazole in complex is better than the pure drug, and it will take a longer time for the pure drug to dissolve. Therefore, *T*_max_ in the complex group will be shorter than that of the pure drug group. Since the blood drug concentration in the complex group was higher than that in the pure drug group, it is no wonder that the half-life and MRT of the drug in the complex group were longer than those in the pure drug group.

## Materials and experiments

Methyl β-cyclodextrin (DS: 11–13), HP β-cyclodextrin (average Mw: 1460), HP γ-cyclodextrin (DS: 4–6), β-cyclodextrin, and γ-cyclodextrin were purchased from Shanghai McLean Biochemical Technology Co., Ltd. (Shanghai, P. R. China). Mebendazole (≧99%) was purchased from Beijing Solebao Technology Co., Ltd. (Beijing, P. R. China). Formic acid, acetonitrile, and methanol were obtained from Xilong Science Co., Ltd. (Guangzhou, P. R. China). The dogs were purchased from Guangdong Medical Laboratory Animal Center.

### Preparation of the solution of mebendazole

Mebendazole (10 mg) in a 10 mL volumetric flask had a small amount of formic acid (1 mL) added, then it was vortexed and pipetted with water (9 mL) to provide a solution with a concentration of 1 mg/mL.

### UV spectral screening of the standard solution of mebendazole

The standard mebendazole solution (1 mg/mL) was vortexed, filtered through a membrane into a cuvette, and scanned using a UV spectrophotometer (from Shanghai Jinghua Technology Instrument Co., Ltd.) in the wavelength range of 200–400 nm at room temperature.

### HPLC standard curve and regression equation establishment

A solution of mebendazole (1 mg) in formic acid (1 mL) was diluted with water to obtain a series of samples with concentrations of 0.01 mg/mL, 0.02 mg/mL, 0.04 mg/mL, 0.06 mg/mL, 0.08 mg/mL and 0.1 mg/mL for HPLC analysis. An LC-15C high-performance liquid chromatograph (from Shimadzu Enterprise Management, China Co., Ltd.) was used to analyze the samples, with a UV detector at 234 nm on a Shimpack VP ODS C18 column (250 × 4.6 mm). The mobile phase was acetonitrile and water (50:50), the flow rate was 1.0 mL/min, and the testing temperature was 30°C. Based on the peak areas in HPLC and the concentrations of mebendazole in solutions, the standard curve (shown in Figure 2) and a linear regression equation were obtained.

### Phase solution study

The solutions of β-cyclodextrin, γ-cyclodextrin, methyl-β-cyclodextrin, HP-β-cyclodextrin, and HP-γ-cyclodextrin in ultrapure water (10 mL) with concentrations of 5 mmol/L, 10 mmol/L, 15 mmol/L, 20 mmol/L, 25 mmol/L, and 30 mmol/L had extra mebendazole added in a shaker at 25°C in the dark, and were shaken for 48 h. After filtration, 1 mL of each reaction was taken for HPLC analysis to provide the phase solution study results (shown in Figure 3).

### Water solubility determination of mebendazole in complex

An extra mebendazole complex was added to water (1 mL) while shaking. After 2 h, the mixture was filtered and analyzed by HPLC to obtain the water solubility of mebendazole in the complex.

### Determination of the inclusion rate and inclusion yield

The formulas shown below were used to calculate the inclusion rate and yield of mebendazole in complexes:


$$\text{Inclusion yield}\;(\%)=[\begin{array}{l} \text{complex}\;(\mathrm{mg})/\text{mebendazole}\;(\mathrm{mg})\\ +\text{cyclodextrin}\;(\mathrm{mg}) \end{array}]\;x\;100\%.$$

$$\text{Inclusion ratio}\;(\%)=[\begin{array}{l} \text{mebendazole in complex}\;(\mathrm{mg})\\ /\text{mebendazole}\;(\mathrm{mg}) \end{array}]\;x\;100\%.$$

### Preparation of the complex of mebendazole with HP-beta-cyclodextrin

HP-beta-cyclodextrin (365.4 mg, 0.25 mmol) was slowly added to the solution of mebendazole (74 mg, 0.25 mmol) in formic acid (8 mL), stirred at 500 r/min at 50°C for 3 h, then cooled in a refrigerator at 4°C for 8 h, evaporated to remove the formic acid (until no formic acid smell), dissolved into water (50 mL), filtered through a 0.22 μm filter membrane, cooled at −20°C overnight, and freeze-dried under reduced pressure to obtain the inclusion complex as a solid powder.

### Preparation of the physical mixture of mebendazole and HP-beta-cyclodextrin

The grounded mebendazole (74 mg, 0.25 mmol) was mixed with grounded HP-beta-cyclodextrin (365.4 mg, 0.25 mmol) and passed through an 80-mesh sieve to provide the physical mixture of mebendazole and HP-beta-cyclodextrin.

### Scanning electron microscopy

Mebendazole, the physical mixture of HP-beta-cyclodextrin and mebendazole, and the inclusion complex were gold-plated, placed on the sample stage, and scanned with a scanning electron microscope (Merlin from the German Zeiss company) with 5 kV accelerating voltage.

### NMR study

The proton NMR spectrum of mebendazole complex with HP-beta-cyclodextrin in D_2_O was recorded using a Bruker 400 NMR spectrometer (from Germany), and the ROESY spectrum was recorded using a Bruker 600 NMR spectrometer (from Germany).

### The dissolution rate study

Based on the paddle method in the first part of the 2010 edition of the Chinese Veterinary Pharmacopeia, the release cups containing ultrapure water (900 mL) degassed by an ultrasonic cleaning machine were placed with mebendazole (50 mg), the complex of mebendazole and HP-β-cyclodextrin (contained 50 mg of mebendazole), and the physical mixture of mebendazole and HP-beta-cyclodextrin (containing 50 mg of mebendazole), and stirred at speed of 100 r/min at 37 ± 0.5°C, respectively. Samples (1 mL) were taken at 1, 3, 5, 15, 30, 45, 60, and 75 min from each cup and filtered through a 0.22 μm microporous filter membrane within 30 s for HPLC analysis, at the same time, 1 mL of the isothermal dissolution medium was added to the cup. Each experiment was repeated three times, and based on the HPLC results, the time-cumulative dissolution curve was obtained and shown in Figure 6.

### *In vivo* PK study

#### Establishment of the standard curve of mebendazole in blank dog plasma

Mebendazole (10 mg) was dissolved into chromatographic pure acetonitrile (2 mL), diluted with chromatographic pure methanol, and added to tubes containing 0.2 mL of blank dog plasma (received from Guangzhou Rui-Te Co., Ltd., Guangzhou, Guangdong, China) to obtain the solutions with concentrations of mebendazole as 0.015 μg/mL, 20 μg/mL, 40 μg/mL, 60 μg/mL, and 80 μg/mL for HPLC analysis. The mobile phase was a gradient of 0.3% potassium phosphate solution and acetonitrile, and the pH of the mobile phase was adjusted to 4 by adding triethylamine. The wavelength of the UV detector was 234 nm, the column temperature was 35°C, the flow rate was 1 mL/min, the injection volume was 50 μL, and gradient elution was as follows: 0–14 min, 25% acetonitrile, 14–18 min, 40% acetonitrile, 18–25 min, 25% acetonitrile. Under these HPLC conditions, the dog plasma did not interfere with the detection of mebendazole. Based on the HPLC data, the standard working curve of mebendazole in blank dog plasma and the regression equation were obtained, as shown in Figure 7.

#### Recovery rate of mebendazole from the dog plasma and intra-day and inter-day precision and accuracy

An appropriate volume of the mebendazole standard solution was added to 500 μL of dog blank plasma to prepare the samples with different concentrations of mebendazole: 10 μg/mL, 1 μg/mL and 0.1 μg/mL. After extraction and filtration, the samples were analyzed by HPLC. Five parallel trials were performed at each concentration, three samples of each concentration were prepared, the intra-day sample was measured three times, and the intra-day difference coefficient was calculated. The test was carried out continuously for 3 days, and the coefficient of inter-day variance was calculated. The recovery rates were obtained in the range of 110% ± 0.25 to 90.8% ± 0.27%. The intra-day difference coefficient was 1.14% ~ 5.67%, and the inter-day difference coefficient was 1.4% ~ 3.5%.

#### Blood sample collection

A total of 12 healthy adult dogs (5 ± 0.1 kg). The group consisted of an equal number of male and female dogs (sourced from Guangdong Medical Laboratory Animal Center, Guangzhou, Guangdong, China). We informed the center that these dogs would be used for an *in vivo* PK study. The dogs were randomly divided into two groups, named as mebendazole group and the inclusion group. They were weighed, numbered, and fed a diet of low-fat dog food (obtained from Shanghai Jibai Chong Industrial Co., Ltd., Shanghai, China) for 1 week in a warm and ventilated environment. Prior to administration, the dogs were fasted for 12 h. In addition, they were dosed orally with mebendazole or its complex at a dosage of 5 mg/kg, and they had access to normal drinking water during sampling. Blood samples (2 mL) were taken from the forearm vein at 0.25, 0.5, 0.75, 1, 2, 4, 6, 10, 12, and 24 h after administration. The samples were immediately transferred to heparinized tubes, centrifuged at 3500 rpm for 10 min, and stored in a freezer at −20°C.

#### Analysis of the blood samples

The plasma sample was thawed at room temperature, and 0.5 mL was pipetted into a 2 mL centrifuge tube. A mixture of dichloromethane and methanol (1:1, 1 mL) was added, and the solution was vortexed for 2 min. It was then centrifuged at a speed of 13,000 r/min for 10 min and transferred to a test tube. The procedure was repeated twice. The resulting solution was evaporated to dryness under a nitrogen stream at 40°C. Following this, 0.5 mL of acetonitrile was added, and the mixture was vortexed for 5 min. It was then centrifuged at 13000 r/min for 10 min, and filtered through a 0.22 μm microporous filter to prepare the sample for HPLC analysis.

### Statistical analysis

All analyses were carried out in triplicate, and statistical analysis was performed using one-way analysis of variance, followed by a significant difference test using Prism 9 software. *p* ≤ 0.05 indicates that the difference is statistically significant.

## Conclusion

Using cyclodextrin and complexation condition selections based on single factors and orthogonal strategies, an inclusion complex of mebendazole/HP-beta-cyclodextrin with a water solubility of 6.05 mg/mL was prepared and confirmed by SEM and NMR spectra. In dissolution rate studies, 80% of mebendazole was released from the complex in 5 min. In an *in vivo* PK study, the bioavailability increased by 91%, indicating that the prepared mebendazole/HP-beta-cyclodextrin complex could be used as a potential anti-cancer agent in clinical practice. Additionally, *in vivo* efficacy study, toxicity study, and immunogenicity assessment are in progress.

## Data Availability

The original contributions presented in the study are included in the article/supplementary material, further inquiries can be directed to the corresponding author.

## References

[1] Joffe LS Schneider R Lopes W Azevedo R Staats CC Kmetzsch L. The anti-helminthic compound mebendazole has multiple antifungal effects against *Cryptococcus neoformans*. Front Microbiol. (2017) 8:535. doi: 10.3389/fmicb.2017.00535, PMID: 28400768 PMC5368277

[2] Clarke NE Doi SAR Wangdi K Chen Y Clements ACA Nery SV. Efficacy of anthelminthic drugs and drug combinations against *soil-transmitted helminths*: a systematic review and network meta-analysis. Clin Infect Dis. (2019) 68:96–105. doi: 10.1093/cid/ciy423, PMID: 29788074

[3] Guerini AE Triggiani L Maddalo M Bonù ML Frassine F Baiguini A. Mebendazole as a candidate for drug repurposing in oncology: an extensive review of current literature. Cancers. (2019) 11:1284. doi: 10.3390/cancers11091284, PMID: 31480477 PMC6769799

[4] Meco D Attinà G Mastrangelo S Navarra P Ruggiero A. Emerging perspectives on the antiparasitic mebendazole as a repurposed drug for the treatment of brain cancers. Int J Mol Sci. (2023) 24:1334. doi: 10.3390/ijms24021334, PMID: 36674870 PMC9862092

[5] Pantziarka P Bouche G Meheus L Sukhatme V Sukhatme VP. Repurposing drugs in oncology-mebendazole as an anti-cancer agent. Ecancermedicalscience. (2014) 8:443. doi: 10.3332/ecancer.2014.443.eCollection2014 25075217 PMC4096024

[6] Derayea SM Ali HRH Hamad AA Ali R. Application of silver nanoparticles for the spectrophotometric determination of three benzimidazole anthelmintic drugs in their pharmaceutical preparations. J Appl Pharm Sci. (2017) 7:76–82. doi: 10.7324/JAPS.2017.70209

[7] Mansoori S Fryknäs M Alvfors C Loskog A Larsson R Nygren P. A phase 2a clinical study on the safety and efficacy of individualized dosed mebendazole in patients with advanced gastrointestinal cancer. Sci Rep. (2021) 11:8981. doi: 10.1038/s41598-021-88433-y, PMID: 33903692 PMC8076239

[8] Kelly JD Chevis RAF Goodman HT. Effect of particle size on the anthelmintic efficacy of mebendazole against *Nippostrongylus brasiliensis* in the rat. Int J Parasitol. (1975) 5:275–80. doi: 10.1016/0020-7519(75)90073-9, PMID: 1168627

[9] Pervaiz A Saeed MA Anwar M Mansoor U Shabbir U Khalid DM. Preparation and characterization of self-emulsifying solid dispersions of mebendazole to improve solubility. Int J Phar Integr Health Sci. (2023) 4:1–21. doi: 10.56536/ijpihs.v4i1.77

[10] Chaudhary S Garg T Rath G Murthy RR Goyal AK. Enhancing the bioavailability of mebendazole by integrating the principles solid dispersion and nanocrystal techniques, for safe and effective management of human echinococcosis. Artif Cells Nanomed Biotechnol. (2016) 44:1–6. doi: 10.3109/21691401.2014.1000493, PMID: 25783855

[11] García-Rodriguez JJ de la Torre-Iglesias PM Vegas-Sánchez MC Torrado-Durán S Bolás-Fernández F Torrado-Santiago S. Changed crystallinity of mebendazole solid dispersion: improved anthelmintic activity. Int J Pharm. (2011) 403:23–8. doi: 10.1016/j.ijpharm.2010.10.002, PMID: 20934497

[12] Anasuya P Kumar S. Formulation and evaluation of solid dispersions of an anthelmintic drug for enhancement of dissolution rate. J Innovat Pharm Biol Sci. (2017) 4:71–4.

[13] Sandulovici R Mircioiu C Voicu V Ghafil F Mati E Anuta V. Modelling of release kinetics of mebendazole fromsolid dispersion-based formulations in simulted gastric fluid. Rom Biotechnol Lett. (2020) 25:1473–81. doi: 10.25083/rbl/25.2/1473.1481

[14] Ángeles PM Begoña E Guillermo T Paola N. Physicochemical characterization and solubility enhancement studies of mebendazole solid dispersions in solvent mixtures. J Pharm Pharmacol. (2016) 4:351–8. doi: 10.17265/2328-2150/2016.07.009

[15] Roxana S Aboul-Enein HY Voice V Ghafil AF Mati E Anuta V. Determination and modeling of in vitro release kinetics of mebendazole in simulated intestinal fluid from solid dispersion formulations as infinite reservoir. Acta Pol Pharm. (2020) 77:849–61. doi: 10.32383/appdr/131112

[16] Chiba Y Kohri N Iseki K Miyazaki K. Improvement of dissolution and bioavailability for mebendazole, an agent for human echinococcosis, by preparing solid dispersion with polyethylene glycol. Chem Pharm Bull. (1991) 39:2158–60. doi: 10.1248/cpb.39.2158, PMID: 1797442

[17] Yellanki SK Palsodkar MK Deb SK Sharada G Nerella NK. Formulation, dissolution characterization and in-vitro anthelmintic activity of several fast-release solid dispersions of mebendazole. J Pharm Res. (2010) 3:1288–92.

[18] de la Torre-Iglesias PM García-Rodriguez JJ Torrado G Torrado S Torrado-Santiago S Bolás-Fernández F. Enhanced bioavailability and anthelmintic efficacy of mebendazole in dispersible microparticles with low-substituted hydroxypropyl cellulose. Drug Des Devel Ther. (2014) 18:1467–79. doi: 10.2147/DDDT.S65561 PMC4174045 25258515

[19] Kállai-Szabó B Sinka M Stiedl B Sebe I Antal I Zelkó R. Tracking of the solubility enhancement and drug release stability of melt extrudates containing mebendazole. J Drug Deliv Sci Technol. (2014) 24:514–8. doi: 10.1016/S1773-2247(14)50097-4

[20] Rajeshri DP Akanksha SP Henis JP Sruthi S Ketan P. Development of rapidly soluble mebendazole nanosuspension for colorectal cancer. J Drug Deliv Sci Technol. (2024) 91:105276. doi: 10.1016/j.jddst.2023.105276

[21] Swamy NGN Rupa V Abbas Z Dasankoppa FS. Formulation and evaluation of nanosuspensions for enhancing the dissolution of poorly soluble mebendazole. Indian Drugs. (2010) 47:47–54.

[22] Rao MRP Raut SP Shirsath CT Jadhav MB Chandanshive PA. Self-nanoemulsifying drug delivery system was explored to improve the oral bioavailability and target specificity of mebendazole for treatment of lymphatic worm infestations. Indian J Pharm Sci. (2018) 80:1057–68. doi: 10.4172/pharmaceutical-sciences.1000456

[23] Swati VG Mobeen S Sanjay KG Nihar RK Kiran K Seena KX. Fabrication of mebendazole loaded solid lipid nanoparticles: formulation, optimization, characterization, stabilization, and *in-vitro* evaluation. J Clin Otorhinolaryngol Head Neck Surg. (2023) 27:598–610. doi: 10.1080/21691401.2017.1396996

[24] Gutiérrez EL Souza MS Diniz LF Ellena J. Synthesis, characterization and solubility of a new anthelmintic salt: mebendazole nitrate. J Mol Struct. (2018) 1161:113–21. doi: 10.1016/j.molstruc.2018.02.060

[25] Chen J Wang Z Wu C Li S Lu T. Crystal engineering approach to improve the solubility of mebendazole. CrystEngComm. (2012) 14:6221–9. doi: 10.1039/c2ce25724f

[26] de Paula K Camí GE Brusau EV Narda GE Ellena J. Mebendazole mesylate monohydrate: a new route to improve the solubility of mebendazole polymorphs. J Pharm Sci. (2013) 102:3528–38. doi: 10.1002/jps.23658, PMID: 23897162

[27] Camí GE Brusau EV Narda GE Maggio RM. Dual approach for concomitant monitoring of dissolution and transformation at solid-state. Mebendazole salts case study. J Drug Deliv Sci Technol. (2020) 55:101344. doi: 10.1016/j.jddst.2019.101344

[28] Sumimoto Y Okawa S Inoue T Masuda K Maruyama M Higaki K. Extensive improvement of oral bioavailability of mebendazole, a brick dust, by polymer-containing SNEDDS preparation: disruption of high crystallinity by utilizing its counter ion. Eur J Pharm Biopharm. (2022) 172:213–27. doi: 10.1016/j.ejpb.2022.02.002, PMID: 35134511

[29] Bernard JMB Mora JG Diaz D Yatsimirsky A. Interactions among mebendazole and some cyclodextrins. Influence on solubility and dissolution rate. Eur J Pharm Sci. (1996) 4:S143.

[30] Diaz D Bernard MJB Mora JG Llanos CME. Solubility, 1H-NMR, and molecular mechanics of mebendazole with different cyclodextrins. Drug Dev Ind Pharm. (1999) 25:111–5. 10028428 10.1081/ddc-100102151

[31] Alvarez C Hees TV Piel G Liégeois JF Evrard B Torrado JJ. Preparation of mebendazole HP-beta-cyclodextrin complexes using water-soluble polymers and organic acids. STP Pharm Sci. (2001) 11:439–42.

[32] Minda D Mioc A Banciu C Soica C Racoviceanu R Mioc M. Cyclodextrin dispersion of mebendazole and flubendazole improves *in vitro* antiproliferative activity. PRO. (2021) 9:2185. doi: 10.3390/pr9122185

[33] Buchter V Priotti J Leonardi D Lamas MC Keiser J. Activity of novel oral formulations of albendazole and mebendazole against *Heligmosomoides polygyrus in vitro* and *in vivo*. J Pharm Sci. (2020) 109:1819–26. doi: 10.1016/j.xphs.2020.02.002 32070702

[34] Skiba ML Coquard A Bounoure F Vérité P Arnaud P Skiba M. Mebendazole complexes with various cyclodextrins: preparation and physicochemical characterization. J Incl Phenom Macrocycl Chem. (2007) 57:197–20. doi: 10.1007/S10847-006-9196-9

[35] Nguyen TN Tran P Choi YE Park J-S. Solid dispersion of mebendazole via surfactant carrier to improve oral bioavailability and in vitro anticancer efficacy. J Pharm Investig. (2023) 53:443–55. doi: 10.1007/s40005-023-00616-z

[36] Ba OM Skiba ML Joudieh S Bounoure F Skiba M. Influence of water-soluble polymers, pH and surfactants on mebendazole solubilization by β-and partially methylated-β-cyclodextrins. NSTI-Nanotech. (2008) 2:386–9.

